# Effects of Chronic *Banisteriopsis caapi* Extract Administration on Fibrillar Amyloid Deposition and Microglial Morphology in Aged PDGFB-APPSwInd Mice

**DOI:** 10.3390/ph19091434

**Published:** 2026-09-10

**Authors:** Isabella Bacci Bustelli, Santhiago Calvelo Graça, Érica Victória dos Santos, Rafael Lanaro, Alessandra Linardi, Ariadiny Lima Caetano

**Affiliations:** 1 Department of Physiological Sciences, Santa Casa de São Paulo School of Medical Sciences (FCMSCSP), São Paulo 01221-020, SP, Brazil; baccibustelliisabella@gmail.com (I.B.B.); santhiagocalvelo@hotmail.com (S.C.G.); schilman.victoria@gmail.com (É.V.d.S.); alelinardi40@gmail.com (A.L.); 2 Faculty of Medical Sciences, State University of Campinas (UNICAMP), Campinas 13083-894, SP, Brazil; rlanaro@unicamp.br

**Keywords:** Alzheimer’s disease, amyloid-β, *Banisteriopsis caapi*, hippocampus, microglia, neuroinflammation, β-carbolines

## Abstract

**Background/Objectives:** *Banisteriopsis caapi* contains β-carboline alkaloids with neuropharmacological and immunomodulatory properties, but their effects during prolonged exposure in established amyloid pathology remain poorly characterized. This study investigated whether chronic intermittent *B. caapi* administration modifies behavioral performance, fibrillar amyloid pathology, and hippocampal microglial alterations in aged PDGFB-APPSwInd mice. **Methods**: Male wild-type (WT) and PDGFB-APPSwInd transgenic (TG) mice aged 15–18 months received *B. caapi* extract (1.5 mL/kg, oral gavage) or vehicle twice weekly for four weeks. Locomotor activity, anxiety-like behavior, and spatial recognition memory were assessed using the open-field, elevated-plus-maze, and object-location tests, respectively. Hippocampal fibrillar amyloid plaques were evaluated by Thioflavin-S staining, and microglial immunoreactivity and morphology were assessed using Iba1 immunofluorescence and morphometric analysis. **Results**: Chronic *B. caapi* administration did not consistently modify locomotor activity, anxiety-like behavior, or spatial recognition memory. In TG mice, treatment increased the number of hippocampal Thioflavin-S-positive fibrillar amyloid plaques, without corresponding changes in total Thioflavin-S-positive area or median plaque size. Microglial alterations were predominantly genotype-associated, with TG mice exhibiting changes in Iba1-labeled cell quantification and morphology, particularly in the CA1 region. *B. caapi* treatment did not significantly modify these genotype-associated morphological alterations. **Conclusions:** Chronic intermittent *B. caapi* exposure was associated with a selective change in fibrillar amyloid plaque number without parallel changes in plaque area, plaque size, behavioral performance, or microglial morphology. These findings expand current evidence regarding the biological effects of repeated *B. caapi* exposure in the context of established amyloid pathology.

## 1. Introduction

Alzheimer’s disease (AD) is characterized by age-related cognitive and functional decline, along with a specific neuropathology. This refers to two synergistic pathological processes that characterize AD histologically: the accumulation of amyloid-β (Aβ) plaques around neurons and the hyperphosphorylation of the TAU protein. Together, these two processes lead to synaptic loss and neurodegeneration [1].

Although the pathophysiology of AD has not been fully understood to date, the most widely accepted hypothesis is the “amyloid cascade hypothesis”, in which everything starts due to an erroneous cleavage of the amyloid precursor protein (APP), generating amyloid-beta (Aβ) plaques [2]. APP is a transmembrane protein present in all cell types in the body and can be cleaved by two enzymes: α-secretase or a combination of β-secretase and γ-secretase. In the amyloidogenic pathway, APP is cleaved by β-secretase and γ-secretase, producing Aβ peptides, which are difficult to clear [1]. In this sense, an imbalance between production, clearance and aggregation of peptides causes Aβ to accumulate, and this excess can be the triggering factor for AD [2]. Aβ accumulation is associated with downstream pathological events, including tau hyperphosphorylation, neuroinflammation, oxidative stress, and excitotoxicity. Hyperphosphorylated tau progressively aggregates intracellularly into neurofibrillary tangles (NFTs), which initially emerge in transentorhinal regions and subsequently spread to limbic and neocortical areas as the disease progresses. The accumulation and propagation of tau pathology are associated with progressive neuronal dysfunction and loss, ultimately contributing to the emergence of cognitive symptoms [1]. Transgenic models overexpressing human APP carrying familial AD-associated mutations provide robust, age-dependent amyloid pathology and are useful for investigating mechanisms related to amyloid deposition; however, they reproduce selected aspects of familial amyloid-driven disease rather than the full multifactorial complexity of sporadic AD [3,4].

Several studies have demonstrated that microglia are essential for the degradation and removal of Aβ through the regulation of phagocytic processes. These cells are distributed throughout the brain and spinal cord, representing approximately 12–15% of all cells within the CNS [5,6]. Microglia are activated by different types of ligands, mainly pattern recognition receptors (PRRs), including Toll-like receptors (TLRs), as well as other receptors such as CD-14, CD-33, CD-34, RAGE or TREM-2 [7]. 

In response to changes in the brain microenvironment, microglia undergo dynamic transcriptional, functional, and morphological adaptations rather than adopting strictly dichotomous M1/M2 activation states. Current evidence supports a spectrum of context-dependent microglial states whose molecular and functional profiles vary across homeostasis, aging, injury, and neurodegenerative disease [8]. In AD, these heterogeneous responses may influence Aβ recognition and uptake, plaque organization, inflammatory signaling, and tissue homeostasis [9,10]. Persistent dysregulation of these processes may contribute to neuroinflammation and disease progression.

Ayahuasca is a traditional beverage widely consumed in Latin America in religious and holistic rituals. It is primarily prepared from the vines of *Banisteriopsis caapi* (*B. caapi*) and the leaves of *Psychotria viridis* (*P. viridis*) [11]. While *P. viridis* contains the psychedelic compound dimethyltryptamine (DMT), *B. caapi* is rich in the β-carbolines harmine, harmaline, and tetrahydroharmine [12], compounds described as inhibitors of both monoamine oxidase isoforms A and B (MAO-A and MAO-B) [11].

The pharmacological potential of *B. caapi* extract and its major alkaloids has been increasingly investigated, especially in neurological and psychiatric disorders such as Parkinson’s disease and depression [13]. Experimental studies have investigated *B. caapi* extracts in neurodegenerative conditions, although most available evidence has focused on Parkinson’s disease [11,14]. In addition to their neuropharmacological actions, *B. caapi*-derived compounds have been reported to modulate immunological and inflammatory signaling in experimental systems [15,16].

Among the principal alkaloids present in *B. caapi*, β-carbolines such as harmine, harmaline, and tetrahydroharmine have been associated with antidepressant, antioxidant, and antigenotoxic activities, in addition to their inhibitory actions on MAO-A and MAO-B, enzymes involved in catecholamine metabolism [17,18,19]. Furthermore, β-carbolines have been described as inverse agonists of γ-aminobutyric acid (GABA) GABAA receptors, and they also modulate 5-HT2A and 5-HT2C receptors, increasing dopamine efflux [20].

Evidence has also highlighted the anti-inflammatory effects of these compounds. Santos and colleagues (2022 [15]) demonstrated that β-carbolines derived from *B. caapi* exert concentration-dependent effects in BV-2 microglial cells. Higher concentrations were associated with cytotoxicity, whereas lower concentrations significantly reduced the release of pro-inflammatory cytokines. Harmaline, in particular, decreased the secretion of nearly all evaluated cytokines, except IL-6 [15]. In parallel, harmine has been shown to inhibit NF-κB activation induced by lipopolysaccharide (LPS) and TNF-α, while reducing the expression of IL-1β, TNF-α, and IL-6 both in vivo and in vitro in RAW 264.7 macrophage cells [21]. Consistently, Jin et al. (2022) reported that harmine suppressed inducible NOS, COX-2, TNF-α, IL-6, IL-12, and other inflammatory mediators in LPS-stimulated BALB/c and C57BL/6 mouse macrophages [22].

Although *B. caapi* alkaloids have shown anti-inflammatory effects in cell-based and acute experimental settings, their consequences during chronic exposure and established amyloid pathology remain insufficiently characterized. We therefore tested the hypothesis that chronic *B. caapi* administration would modify hippocampal Iba1-labeled cells and its morphology, as well as fibrillar amyloid burden, in aged PDGFB-APPSwInd mice and examined whether these neuropathological effects were accompanied by changes in locomotor activity, anxiety-like behavior, or spatial memory.

## 2. Results

### 2.1. Effects of Chronic B. caapi Treatment on Body Weight and Locomotor Performance

**Figure 1:**
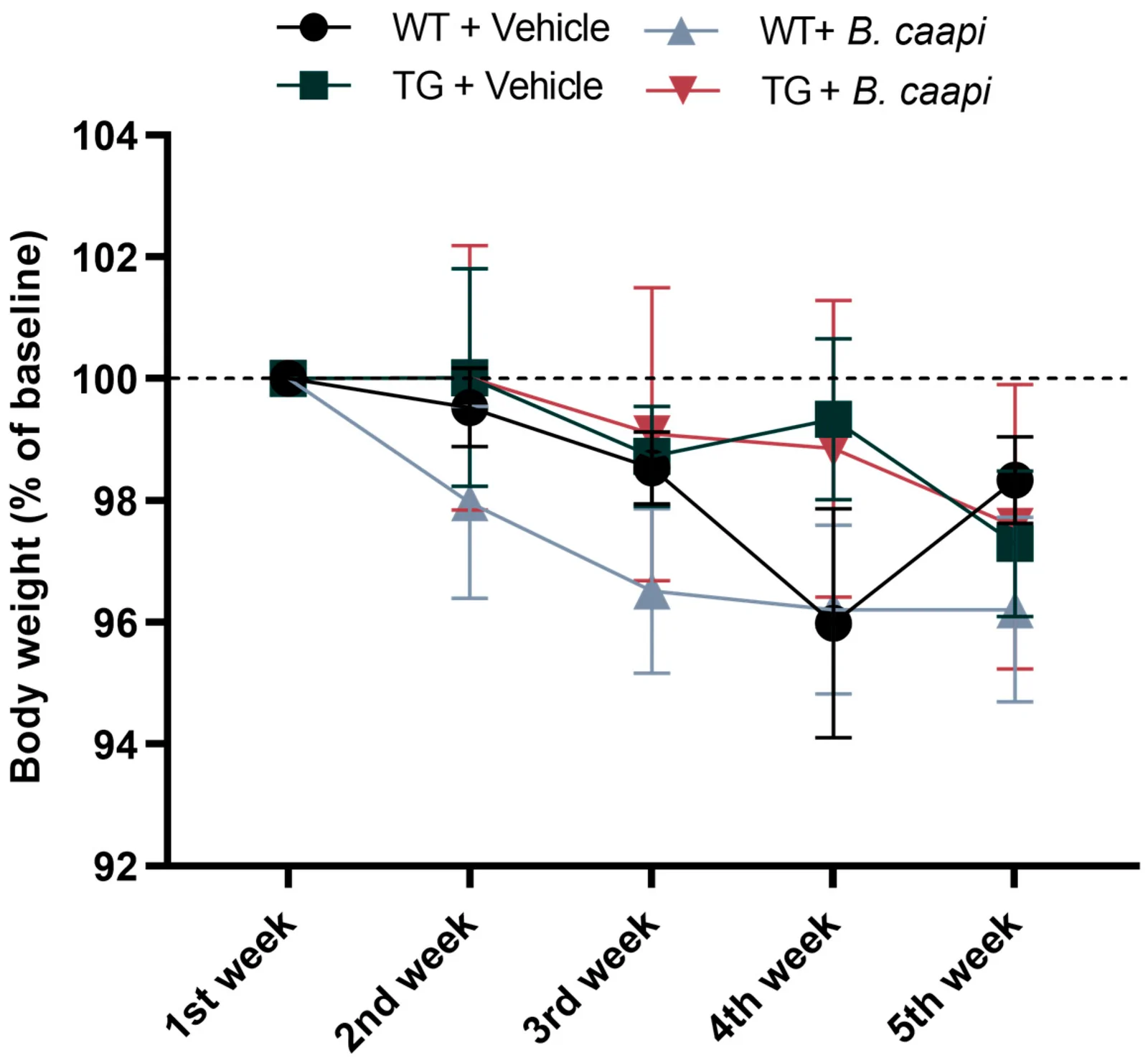
Body weight expressed as the percentage of baseline body weight (Week 1 = 100%) throughout the experimental period. Data are presented as mean ± SEM (N = 8–10 animals/group). Statistical analysis was performed from Week 2 onward using a linear mixed-effects model.

Body weight, expressed as a percentage of baseline body weight, was monitored throughout the experimental period (Figure 1). A linear mixed-effects model revealed a significant main effect of time (F(3,93) = 6.28, *p* = 0.0006), whereas no significant main effects of genotype (F(1,31) = 0.98, *p* = 0.3295) or treatment (F(1,31) = 0.21, *p* = 0.6531) were observed. Likewise, no significant interactions were detected between genotype and treatment (F(1,31) = 0.23, *p* = 0.6334), genotype and time (F(3,93) = 2.60, *p* = 0.0566), treatment and time (F(3,93) = 0.25, *p* = 0.8600), or among genotype, treatment, and time (F(3,93) = 1.03, *p* = 0.3850). Post hoc Sidak’s multiple-comparisons test showed that body weight at weeks 4 and 5 differed significantly from week 2 (*p* = 0.0044 and *p* = 0.0009, respectively), while no other pairwise comparisons reached statistical significance. These findings indicate that body weight changed over time independently of genotype or chronic *B. caapi* treatment.

**Figure 2:**
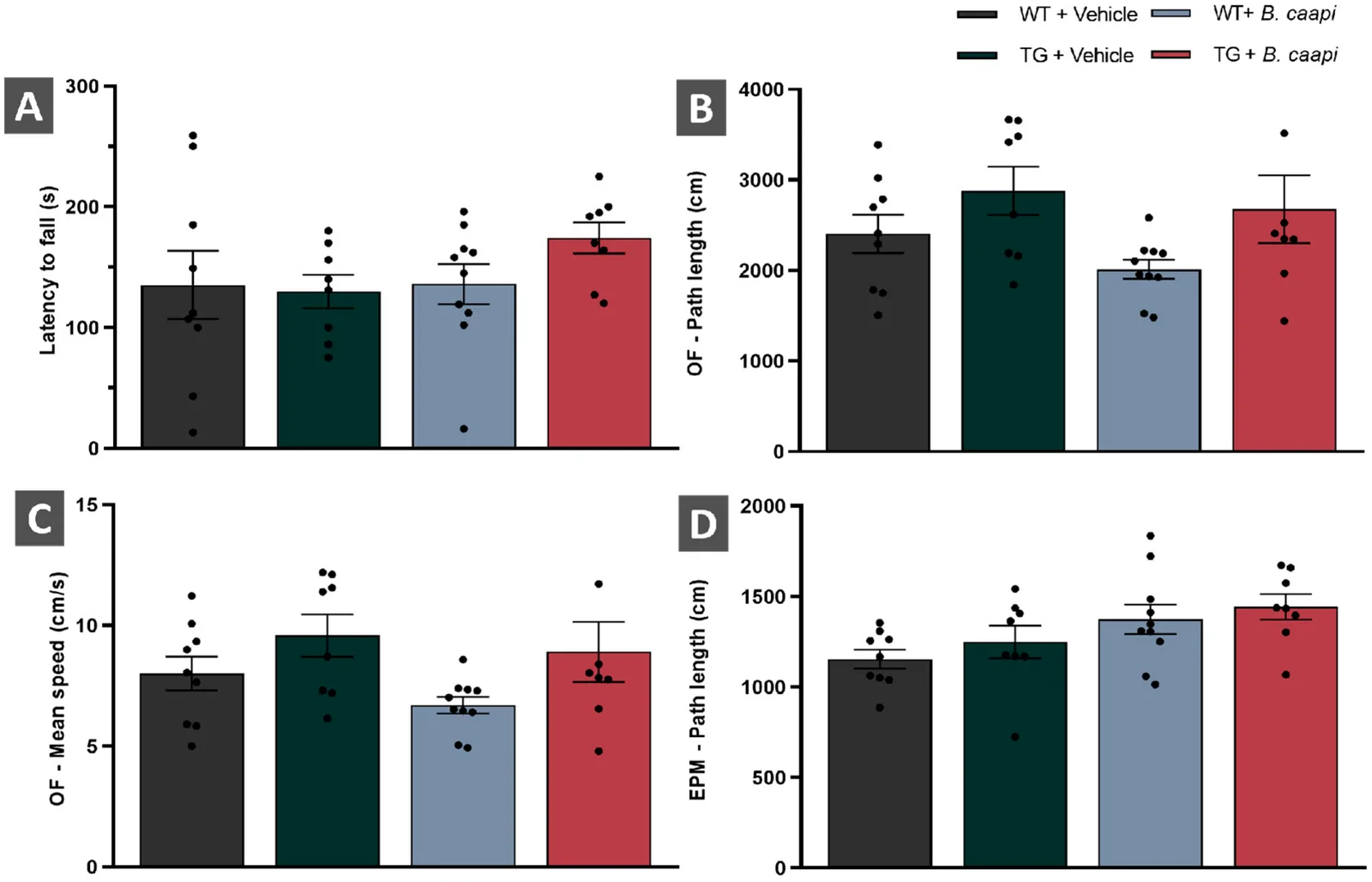
Motor performance assessed in the behavioral tests. (**A**) Latency to fall in the rotarod test (s), (**B**) total distance traveled in the Open Field (OF) test (cm), (**C**) mean speed in the Open Field test (cm/s), and (**D**) total distance traveled in the Elevated Plus Maze (EPM) test (cm). Data are presented as mean ± SEM (N = 8–10 animals/group). Statistical analyses were performed using two-way ANOVA with genotype (WT vs. TG) and treatment (vehicle vs. *B. caapi*) as fixed factors. Sidak’s multiple-comparisons test was applied when appropriate.

To investigate whether genotype and chronic *B. caapi* treatment affected motor performance, mice were evaluated using the rotarod, Open Field (OF), and Elevated Plus Maze (EPM) tests (Figure 2). In the rotarod test, two-way ANOVA revealed no significant genotype × treatment interaction (F(1,31) = 1.263, *p* = 0.2697) and no main effects of genotype (F(1,31) = 0.700, *p* = 0.4092) or treatment (F(1,31) = 1.341, *p* = 0.2557), indicating that neither the PDGFB-APPSwInd genotype nor chronic *B. caapi* treatment affected motor coordination (Figure 2A).

Similarly, no significant genotype × treatment interaction was observed for either the total distance traveled (F(1,31) = 0.1516, *p* = 0.6997) or mean speed (F(1,31) = 0.1532, *p* = 0.6982) in the Open Field test. However, significant main effects of genotype were detected for both total distance traveled (F(1,31) = 5.433, *p* = 0.0264) and mean speed (F(1,31) = 5.421, *p* = 0.0266), whereas no significant main effects of treatment were observed (distance: F(1,31) = 1.482, *p* = 0.2327; speed: F(1,31) = 1.485, *p* = 0.2322), indicating a significant effect of genotype on Open Field performance but not by chronic *B. caapi* treatment (Figure 2B,C).

In the Elevated Plus Maze, no significant genotype × treatment interaction (F(1,31) = 0.0292, *p* = 0.8655) or main effect of genotype (F(1,31) = 1.178, *p* = 0.2860) was detected. A significant main effect of treatment was observed (F(1,31) = 7.578, *p* = 0.0098), indicating that *B. caapi* treatment influenced the overall distance traveled in the apparatus irrespective of genotype (Figure 2D). Although significant main effects were detected for some behavioral parameters, Sidak’s multiple-comparisons test did not reveal significant pairwise differences between individual experimental groups.

### 2.2. Chronic B. caapi Treatment Does Not Modulate Genotype-Dependent Alterations in Anxiety-like Behavior

**Figure 3:**
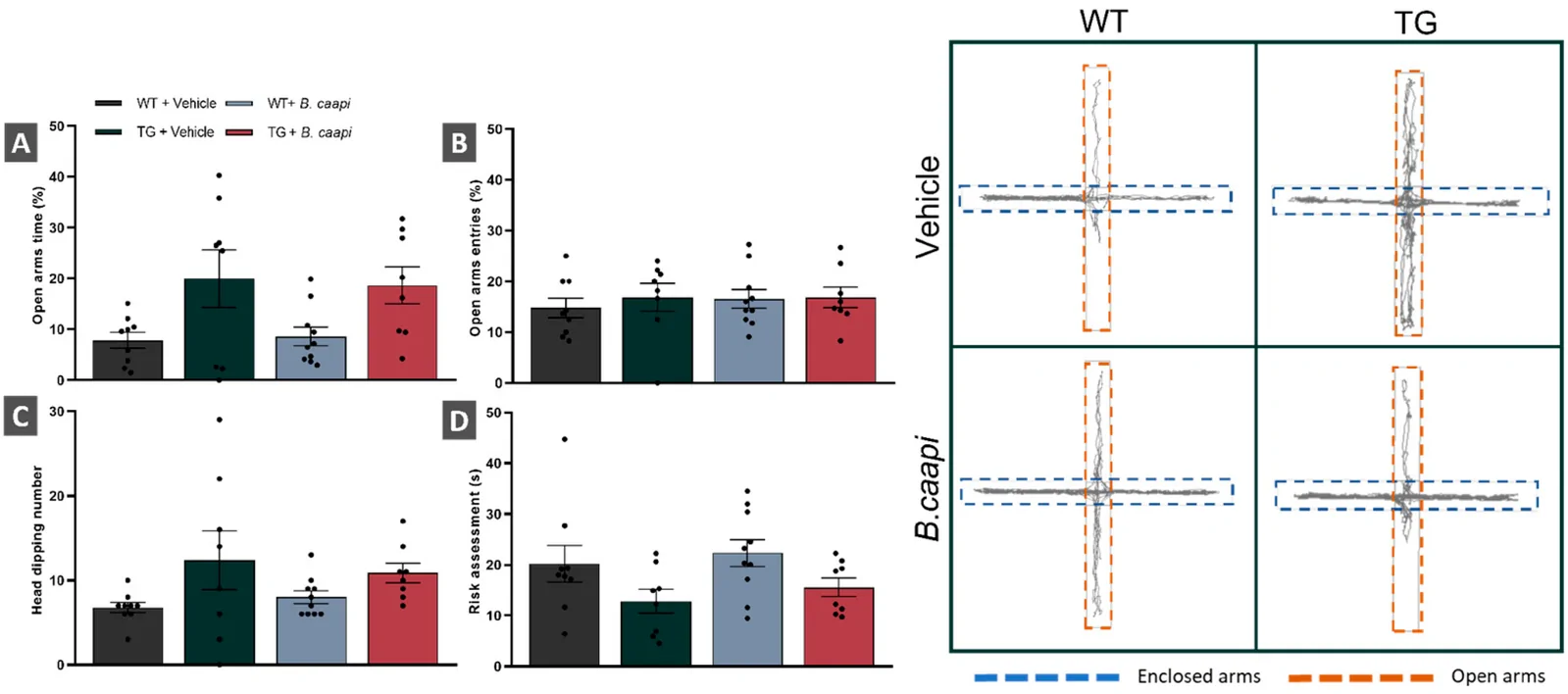
Anxiety-like behavior parameters evaluated in elevated plus maze apparatus. (**A**) Percentage of time spent in open arms of the EPM, (**B**) percentage of entries into the open arms of EPM, (**C**) Number of head dips in the EPM, (**D**) risk assessment (in seconds) in EPM apparatus. Two-way ANOVA (N = 8–10 animals/group).

Regarding anxiety-like behavior, the EPM test revealed a significant main effect of genotype on open arm time (F(1,31) = 10.93, *p* = 0.0024), with TG animals spending more time in the open arms compared with WT animals, whereas no significant effect of treatment (F(1,31) = 0.0085, *p* = 0.9269) or genotype × treatment interaction (F(1,31) = 0.0965, *p* = 0.7582) was observed (Figure 3A). No significant effects of genotype, treatment, or interaction were detected for open arm entries (genotype: F(1,31) = 0.3206, *p* = 0.5753; treatment: F(1,31) = 0.1752, *p* = 0.6784; interaction: F(1,31) = 0.1849, *p* = 0.6701) (Figure 3B).

Analysis of exploratory behaviors showed a significant genotype effect on head dipping number (F(1,31) = 5.826, *p* = 0.0219), while treatment and genotype × treatment interaction were not significant (Figure 3C). Similarly, risk assessment behavior was affected by genotype (F(1,31) = 6.475, *p* = 0.0161), without significant effects of treatment or interaction (Figure 3D).

In the open field test, the time spent in the central zone was not affected by genotype, treatment, or genotype × treatment interaction, suggesting no alterations in this anxiety-related parameter.

### 2.3. Spatial-Memory Outcomes Are Primarily Influenced by Repeated Testing

**Figure 4:**
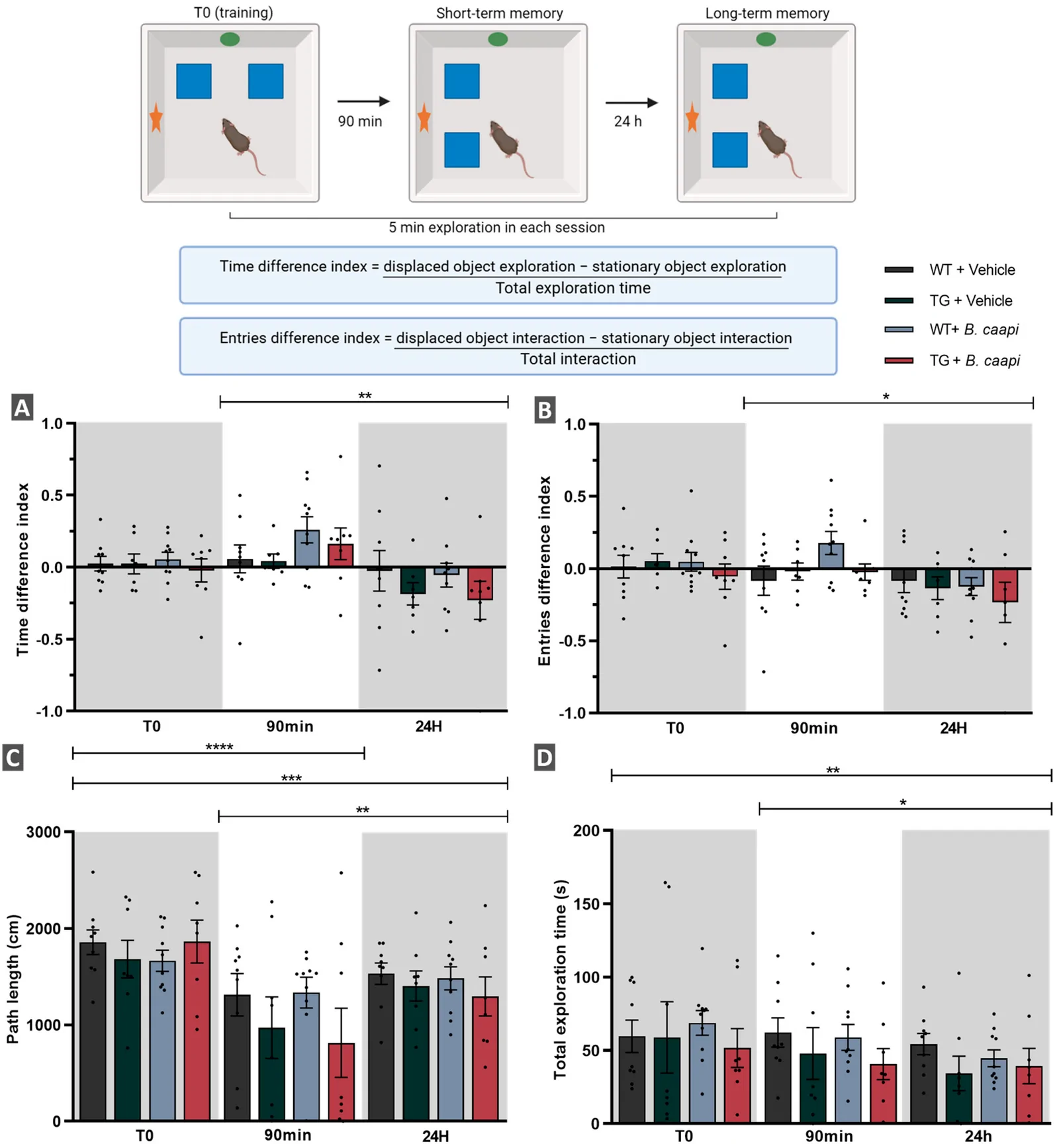
Spatial memory parameters evaluated in object-location test. Graphs show data of T0 (training session), 90 min (short-term memory) and 24 h (long-term memory) of (**A**) time difference index, (**B**) entries difference index, (**C**) path length and (**D**) total exploration time in both objects. Three-way mixed-effects model, with genotype (WT vs. TG) and treatment (vehicle vs. *B. caapi*) as between-subject factors and time (T0, 90 min, and 24 h) as the repeated-measures factor. Significant pairwise comparisons identified by Sidak’s post hoc test (* *p* < 0.05, ** *p* < 0.01, *** *p* < 0.001, **** *p* < 0.0001). (N = 8–10 animals/group). Illustrative diagram of the behavioral test created in BioRender (2026). Calvelo graça, S. https://BioRender.com/l1ih875 (accessed on 12 August 2026).

The object location test assesses the spatial memory of animals by measuring the number of entries at fixed and moving objects, the time spent near each reported object, and the indices generated from these data (Time Difference Index and Entries Difference Index, Figure 4). The parameters mentioned were evaluated 90 min and 24 h after the animals’ first contact with the objects. First, the discrimination index based on exploration time was analyzed (Figure 4A). At baseline (T0), all groups displayed values close to zero, indicating no initial preference for either object location and confirming comparable exploratory behavior before treatment.

Analysis of the time difference index revealed a significant main effect of time (F(1.793, 80.67) = 7.582, *p* = 0.0014). However, no significant effects of treatment (F(1,90) = 0.5230, *p* = 0.4714), genotype (F(1,90) = 2.671, *p* = 0.1057), or any interaction among factors were detected (all *p* > 0.05). Sidak’s post hoc test demonstrated a significant difference between the 90 min and 24 h time points (mean difference = 0.2537, 95% CI = 0.0729–0.4345, *p* = 0.0037), whereas comparisons between T0 and 90 min (*p* = 0.1034) and between T0 and 24 h (*p* = 0.1407) were not significant (Figure 4A).

A similar pattern was observed for the entries difference index. A significant effect of time was detected (F(1.788, 80.44) = 4.862, *p* = 0.0127), while treatment (F(1.90) = 0.0251, *p* = 0.8745), genotype (F(1.90) = 1.588, *p* = 0.2109), and all interaction terms remained non-significant (all *p* > 0.05). Post hoc analysis revealed a significant difference between 90 min and 24 h (mean difference = 0.1622, 95% CI = 0.0055–0.3189, *p* = 0.0407), whereas no differences were observed between T0 and 90 min (*p* = 0.9996) or between T0 and 24 h (*p* = 0.0788) (Figure 4B).

Locomotor activity, assessed as total distance traveled, was also significantly affected by time (F(1.589, 49.26) = 22.92, *p* < 0.0001). No significant effects of treatment (F(1,31) = 0.0956, *p* = 0.7592), genotype (F(1.31) = 1.393, *p* = 0.2468), or interaction terms were observed (all *p* > 0.05). Sidak’s post hoc analysis showed that total distance traveled was significantly reduced at 90 min compared with T0 (mean difference = 637.0, 95% CI = 332.3–941.6, *p* < 0.0001) and remained lower at 24 h (mean difference = 329.7, 95% CI = 137.7–521.7, *p* = 0.0004). In addition, locomotor activity was significantly higher at 24 h than at 90 min (mean difference = −307.3, 95% CI = −536.7 to −77.8, *p* = 0.0058) (Figure 4C), consistent with the development of habituation across repeated testing sessions.

Likewise, total exploration time was significantly influenced by time (F(1.630, 50.54) = 7.733, *p* = 0.0023), whereas no effects of genotype, treatment, or interactions were detected (all *p* > 0.05). Post hoc comparisons revealed a significant reduction in total exploration time at 24 h compared with T0 (mean difference = 16.73, 95% CI = 3.96–29.50, *p* = 0.0070) and compared with 90 min (mean difference = 9.50, 95% CI = 0.10–18.89, *p* = 0.0469). No significant difference was observed between T0 and 90 min (*p* = 0.1390) (Figure 4D).

Collectively, these findings indicate that behavioral changes observed during the object location task were primarily driven by time-dependent effects and were not influenced by either genotype or *B. caapi* treatment.

### 2.4. Chronic B. caapi Treatment Increases Hippocampal Thioflavin-S-Positive Plaque Number in TG Mice

**Figure 5:**
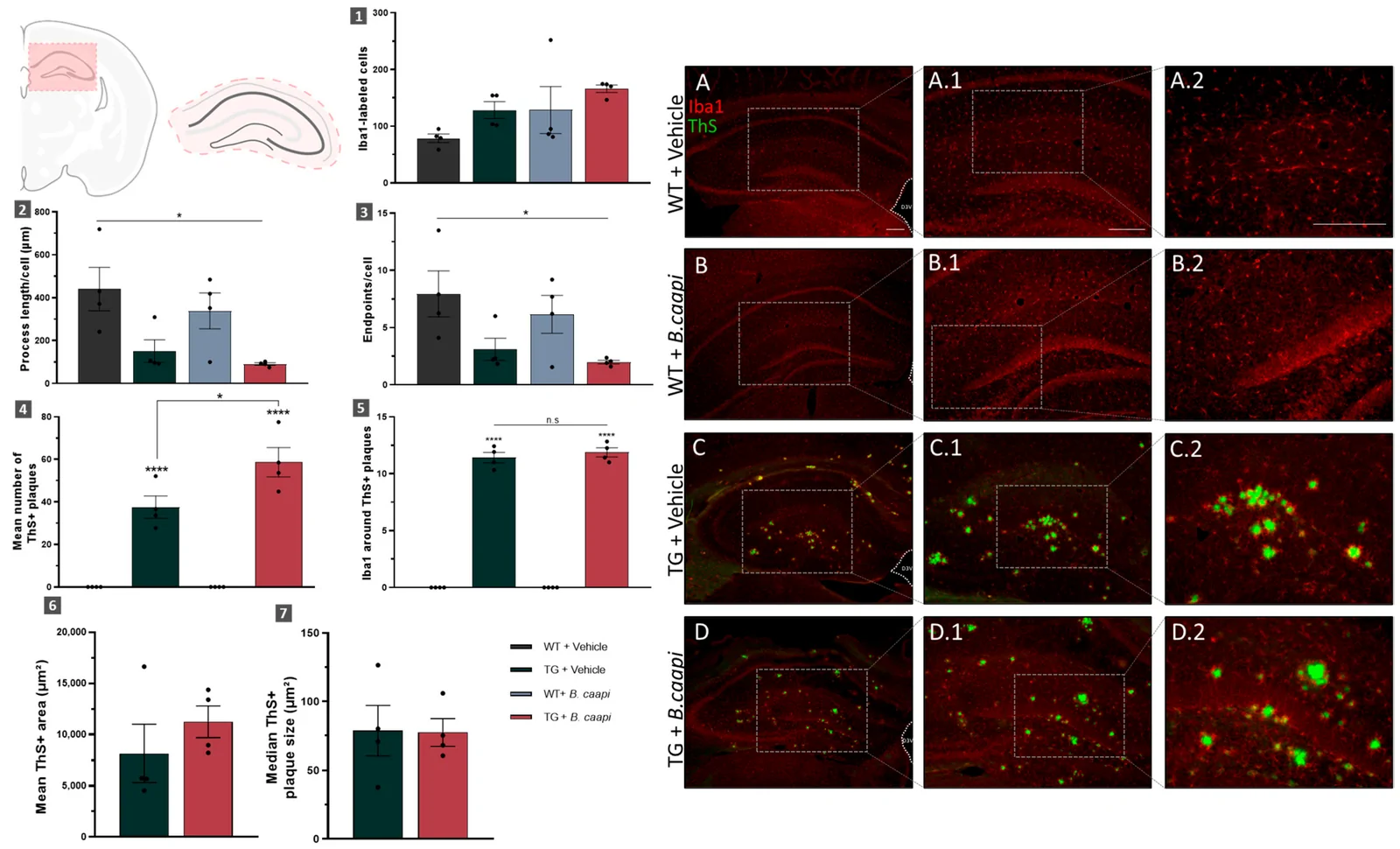
Photomicrographs on the right show cells of dorsal hippocampus labeled with Iba1 (red) and ThS-positive fibrillar amyloid plaques stained with Thioflavin-S (green). (**A**,**A.1**,**A.2**) WT + Vehicle; (**B**,**B.1**,**B.2**) WT + *B. caapi*; (**C**,**C.1**,**C.2**) TG + Vehicle; (**D**,**D.1**,**D.2**) TG + *B. caapi*; (**1**) Iba1-labeled cells. (**2**) The process length/cell (µm) of Iba1-labeled cells. (**3**) Number of endpoints/cell of Iba1-labeled cells. (**4**) Mean number of ThS+ plaques in the dorsal hippocampus. (**5**) Number of Iba1-labeled cells around ThS+ plaques. (**6**) Mean total ThS-positive area (µm^2^) and (**7**) median ThS+ plaque size (µm^2^). (N = 4 animals/group). Two-way ANOVA and total ThS-positive area and animal-level median plaque area were compared between TG + vehicle and TG + *B. caapi* groups using two-tailed unpaired *t*-tests (* *p* < 0.05, **** *p* < 0.0001). ThS, Thioflavin-S. D3V, dorsal third ventricle. Scale bar: 200 μm for all magnifications. Illustrative representation of the hippocampus created in BioRender (2026). Calvelo Graça, S. https://BioRender.com/ayfsbhv (accessed on 12 August 2026).

Thioflavin-S (ThS) staining was performed to evaluate ThS-positive fibrillar amyloid deposition (Figure 5). As expected, no ThS-positive plaques were detected in WT animals, whereas TG mice exhibited marked hippocampal ThS-positive fibrillar amyloid deposition. Two-way ANOVA revealed significant main effects of genotype (F(1,12) = 123.7, *p* < 0.0001) and treatment (F(1,12) = 5.961, *p* = 0.0311), as well as a significant genotype × treatment interaction (F(1,12) = 5.961, *p* = 0.0311) for plaque number. Sidak’s multiple comparisons test showed that TG mice treated with *B. caapi* exhibited a higher number of hippocampal ThS-positive fibrillar amyloid plaques compared with TG vehicle-treated mice (TG + vehicle: 37.52 ± 5.21 vs. TG + *B. caapi* 58.62 ± 6.89 plaques) (*p* = 0.0283). No difference was observed between WT vehicle- and WT *B. caapi*-treated animals (*p* > 0.9999), indicating that the effect of *B. caapi* was restricted to the transgenic phenotype.

In contrast, the mean total ThS-positive area was 8159.09 ± 2841.17 µm^2^ in TG vehicle-treated mice and 11251.37 ± 1541.92 µm^2^ in TG mice treated with *B. caapi*. An unpaired two-tailed *t*-test revealed no significant difference between groups (t = 0.9566, df = 6, *p* = 0.3757). Similarly, the mean of the animal-level median ThS-positive plaque area was 78.76 ± 18.33 µm^2^ in TG vehicle-treated mice and 77.53 ± 9.92 µm^2^ in TG mice treated with *B. caapi*. No significant difference was observed between groups (unpaired two-tailed *t*-test, t = −0.05901, df = 6, *p* = 0.9549). Together, these findings indicate that chronic *B. caapi* treatment increased the number of hippocampal ThS-positive fibrillar amyloid plaques in TG mice without significantly altering the total Thioflavin-S-positive area or the median area of individual plaques.

### 2.5. Microglial Alterations Are Predominantly Associated with Genotype in the Hippocampus

The hippocampus is a highly organized and functionally specialized brain structure that can be subdivided into distinct anatomical regions, each contributing in a specific way to learning and memory processes. These subdivisions are collectively referred to as the Cornu Ammonis (CA) fields and are classically categorized into CA1, CA2, and CA3. Each of these areas possesses unique patterns of connectivity, cellular composition, and physiological properties that support different aspects of hippocampal function [23].

Among these subfields, CA1 is particularly important because it serves as a major output region of the hippocampus. It integrates and processes information, refining and transmitting this information to other brain regions. Due to its strategic connectivity and role in synaptic plasticity mechanisms, CA1 is considered essential for memory consolidation, especially in the formation and stabilization of declarative and spatial memories [24].

In the dorsal hippocampus, two-way ANOVA revealed no significant effects of genotype, treatment, or genotype × treatment interaction on the number of Iba1-labeled cells (genotype: F(1,12) = 3.762, *p* = 0.0763; treatment: F(1,12) = 3.798, *p* = 0.0751; interaction: F(1,12) = 0.07159, *p* = 0.7936), indicating that neither the transgenic phenotype nor chronic *B. caapi* administration significantly altered overall Iba1-labeled cells.

Analysis of microglial morphology revealed genotype-dependent alterations. The number of endpoints was significantly affected by genotype (F(1,12) = 10.53, *p* = 0.0070), whereas no significant effects of treatment (F(1,12) = 1.078, *p* = 0.3195) or genotype × treatment interaction (F(1,12) = 0.05895, *p* = 0.8123) were observed. Similarly, branch length was significantly influenced by genotype (F(1,12) = 14.35, *p* = 0.0026), with no significant effects of treatment (F(1,12) = 1.312, *p* = 0.2744) or interaction (F(1,12) = 0.08304, *p* = 0.7781). These findings indicate that morphological alterations in hippocampal microglia were primarily associated with the transgenic phenotype rather than chronic *B. caapi* exposure.

**Figure 6:**
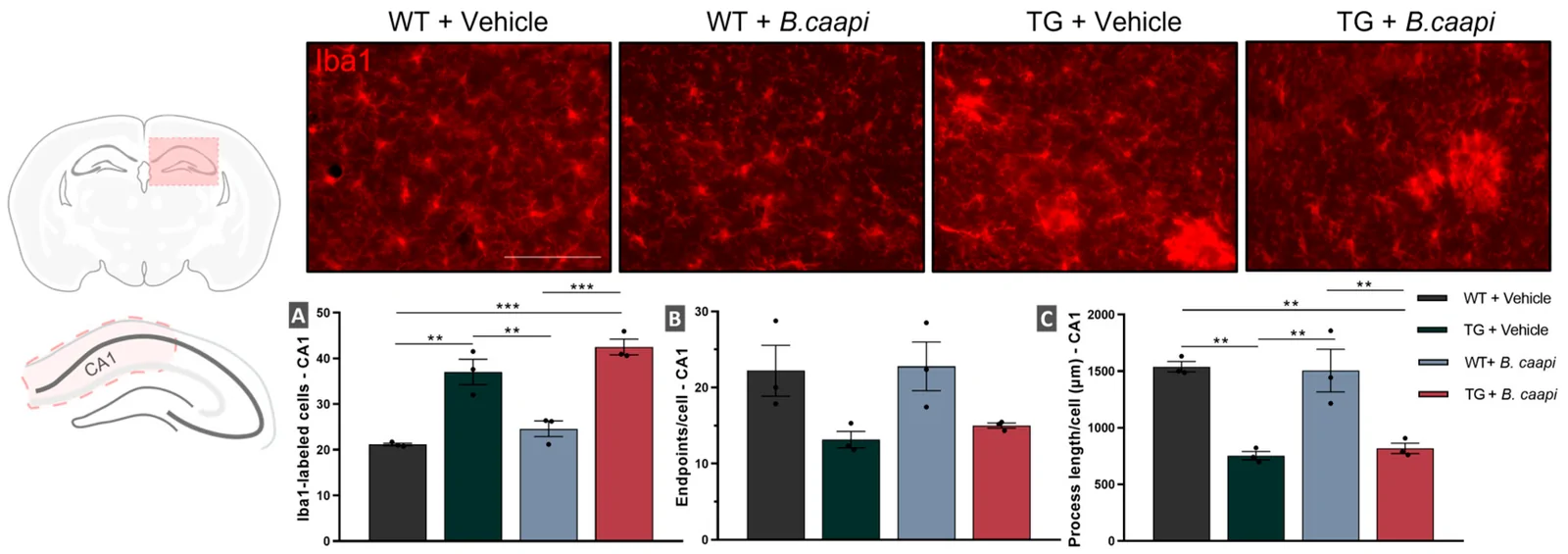
Photomicrographs show microglia of CA1 labeled with Iba1. (**A**) Iba1-labeled cells. (**B**) Number of endpoints/cell of Iba1-labeled cells and (**C**) process length/cell (in micrometers) of Iba1-labeled cells. Two-way ANOVA (** *p* < 0.01, *** *p* < 0.001). CA1, Cornu Ammonis 1 (N = 3 animals/group). Scale bar: 100 μm. Illustrative representation of the hippocampus and CA1 created in BioRender. Calvelo Graça, S. (2026) https://BioRender.com/lfdpq2o (accessed on 12 August 2026).

Considering the relevance of the CA1 subfield in cognitive processes, microglial parameters were further analyzed in this region (Figure 6). Two-way ANOVA demonstrated a robust effect of genotype on Iba1-labeled cells (F(1,8) = 83.94, *p* < 0.0001), with increased microglial labeling in TG animals compared with WT controls. Although a main effect of treatment was detected (F(1,8) = 5.812, *p* = 0.0425), no significant genotype × treatment interaction was observed (F(1,8) = 0.3042, *p* = 0.5963). Sidak’s multiple comparisons test confirmed higher Iba1-labeled cells in TG animals compared with WT groups, while no significant differences were detected between vehicle and *B. caapi*-treated animals within either genotype (WT vehicle vs. WT *B. caapi*, adjusted *p* = 0.7835; TG vehicle vs. TG *B. caapi*, adjusted *p* = 0.3510). Even though a significant overall main effect of treatment was detected, the absence of a genotype × treatment interaction and of significant within-genotype pairwise differences indicates that the overall treatment effect was not specifically attributable to *B. caapi* within the transgenic group. Thus, the increased Iba1-labeled cells in CA1 were predominantly associated with the transgenic genotype rather than with a genotype-specific effect of *B. caapi* treatment.

Morphological analysis in the CA1 region further demonstrated significant genotype-dependent alterations. The number of endpoints was affected by genotype (F(1,8) = 12.50, *p* = 0.0077), while no significant effects of treatment (F(1,8) = 0.2557, *p* = 0.6267) or genotype × treatment interaction (F(1,8) = 0.07009, *p* = 0.7979) were observed. Likewise, branch length was significantly altered by genotype (F(1,8) = 53.16, *p* < 0.0001), with no significant effect of treatment (F(1,8) = 0.02327, *p* = 0.8825) or interaction (F(1,8) = 0.2355, *p* = 0.6405).

Together, these results indicate that hippocampal microglial alterations were predominantly associated with the transgenic phenotype, affecting both Iba1-labeled cells and morphological parameters, particularly within the CA1 region. Chronic *B. caapi* treatment did not significantly modify these genotype-associated morphological changes.

## 3. Discussion

Chronic administration of *Banisteriopsis caapi* extract was evaluated for its effects on behavioral performance, amyloid pathology, and microglial alterations in aged PDGFB-APPSwInd mice. Overall, treatment did not produce consistent alterations in locomotor activity, anxiety-like behavior, or hippocampus-dependent spatial memory. Neuropathological analyses revealed an increased number of hippocampal ThS-positive plaques in *B. caapi*-treated TG mice without significant changes in total ThS-positive area or median plaque size, whereas microglial alterations were predominantly associated with the transgenic phenotype. No significant genotype- or treatment-related differences were detected in the Object Location Test. Because this is an established hippocampus-dependent paradigm for spatial recognition memory [25,26], the present findings indicate that chronic *B. caapi* administration did not significantly modify performance in this specific hippocampus-dependent task under the experimental conditions employed. Behavioral phenotypes in APP-based models may vary with transgenic line, age, disease stage, and behavioral paradigm [27]; therefore, complementary hippocampus-dependent tasks may further extend the behavioral characterization of this model.

A notable neuropathological finding was the higher number of hippocampal ThS-positive plaques in *B. caapi*-treated TG mice, whereas total ThS-positive area and median plaque size were not significantly altered. Thioflavin-S preferentially labels β-sheet-rich fibrillar amyloid aggregates and is widely used to visualize and quantify mature amyloid plaques in APP transgenic models [28,29]. Accordingly, the present data indicate a treatment-associated change in the number of fibrillar amyloid deposits without a parallel increase in the overall area occupied by these deposits or in their median size. This pattern raises questions regarding the dynamics of fibrillar plaque formation, remodeling, and clearance. Complementary approaches, including Aβ-specific immunolabeling and analyses directed at amyloid dynamics, may provide additional insight into the biological processes associated with this observation.

Microglial alterations were predominantly associated with genotype, with TG mice showing reduced branch length and fewer endpoints, particularly in CA1, consistent with morphological remodeling associated with amyloid pathology [8,30]. In this region, increased Iba1-labeled cell quantification further supported genotype-associated remodeling. Although a main effect of treatment was detected for the Iba1-labeled cells, the absence of a genotype × treatment interaction or significant within-genotype differences indicates that this effect was not specifically associated with *B. caapi* treatment within the TG group. Thus, the CA1 findings complement the broader hippocampal pattern and suggest that chronic *B. caapi* exposure did not markedly modify the structural microglial parameters evaluated. Iba1-labeled cell quantification and morphometric assessment provide relevant information on microglial distribution and structural remodeling, while complementary molecular and individual-cell approaches may further characterize the cellular profiles associated with these changes. The focused CA1 analysis involved a smaller number of animals and therefore provides complementary regional information that may be further explored in expanded cohorts.

The biological profile of *B. caapi* and its β-carbolines provides an important context for these observations. Experimental studies have reported modulation of inflammatory signaling and related cellular pathways by *B. caapi*-derived β-carbolines [15,16,31], although findings from cellular and preclinical systems do not necessarily predict therapeutic effects in established AD pathology. Likewise, MAO inhibition represents one component of the pharmacological actions of these compounds rather than a direct predictor of their effects in AD. The twice-weekly regimen used here was designed as chronic intermittent exposure, following a previously published mouse protocol and approximating the non-daily pattern associated with ritual ayahuasca use [32]. Given the relatively short pharmacokinetic profiles reported for β-carboline alkaloids [33], this regimen is more appropriately considered repeated intermittent rather than continuous exposure. Future pharmacokinetic characterization may further clarify the relationship between exposure profile and the biological responses observed.

The scope of these findings should also be considered in relation to the experimental design. The PDGFB-APPSwInd model provides robust age-dependent amyloid pathology and is useful for investigating processes associated with established amyloid deposition, while representing selected aspects of familial amyloid-driven AD rather than the full multifactorial nature of sporadic disease [3,4]. The use of aged male mice defines the experimental population examined, and studies including females may extend these observations given the recognized influence of sex on amyloid pathology and neuroimmune responses [34,35]. Taken together, the present findings provide further insight into the effects of chronic intermittent *B. caapi* exposure in aged mice with established amyloid pathology. While treatment did not consistently modify the behavioral or microglial parameters evaluated, it was associated with an increased number of hippocampal ThS-positive fibrillar plaques in TG mice without corresponding changes in total ThS-positive area or median plaque size, while microglial structural alterations remained predominantly associated with the transgenic phenotype. These observations expand the still limited experimental evidence regarding repeated *B. caapi* exposure in advanced amyloid pathology and highlight the relevance of amyloid plaque characteristics, regional microglial responses, and exposure regimen when interpreting its biological effects. Together, these findings provide a foundation for investigating how responses associated with *B. caapi* exposure may evolve across different stages of amyloid pathology.

## 4. Materials and Methods

### 4.1. Animals

All procedures complied with the ethical principles for animal experimentation established by the Brazilian National Council for the Control of Animal Experimentation (CONCEA) and were approved by the Animal Use Ethics Committee of the Santa Casa de São Paulo School of Medical Sciences (CEUA-FCMSCSP; protocol 006/22). Access to Brazilian genetic heritage was registered in SisGen (A969D82). Animals were obtained from the Department of Physiological Sciences of the Santa Casa de São Paulo School of Medical Sciences and housed in groups of five per cage, with irradiated *Pinus sp*. wood-flake bedding, under controlled environmental conditions (22 ± 1 °C and a 12 h light/dark cycle), with food and water available ad libitum. Environmental enrichment was provided and replaced twice weekly. Animals were monitored throughout the experimental period for general health, behavior, body weight, and signs of distress or adverse effects. No treatment-related adverse events requiring removal of animals from the study were observed. A total of 35 male mice aged 15–18 months were used: 16 PDGFB-APPSwInd transgenic mice and 19 age-matched non-transgenic wild-type (WT) controls. No formal a priori sample-size calculation was performed. The sample size was determined by the number of aged male PDGFB-APPSwInd and age-matched WT mice available that met the predefined age and genotype criteria at the beginning of the study. Animals were allocated to four groups: WT + vehicle (n = 9), TG + vehicle (n = 8), WT + *B. caapi* (n = 10), and TG + *B. caapi* (n = 8). No animals were lost or excluded during the experimental period. The PDGFB-APPSwInd model overexpresses human amyloid precursor protein under the platelet-derived growth factor β-chain promoter and carries the familial AD-associated Swedish (K670N/M671L) and Indiana (V717F) mutations. 

### 4.2. B. caapi Extract Preparation and Administration

*Banisteriopsis caapi* (Spruce ex Griseb.) C.V. Morton extract (World Flora Online; SisGen A969D82) was provided by the Centro Integrado Beneficente Caminho do Vegetal (Hortolândia, SP, Brazil). The lianas were washed, mechanically macerated, boiled in water, and concentrated over several hours to yield approximately 2 L of extract. Based on the quantified alkaloid concentrations in the administered extract and the nominal administration volume of 1.5 mL/kg body weight, each administration corresponded to estimated doses of 0.570 mg/kg tetrahydroharmine, 0.189 mg/kg harmaline, and 0.975 mg/kg harmine. The extract volume was diluted in 40 µL of drinking water immediately before oral gavage; this dilution altered the administered concentration but not the absolute amount of each alkaloid delivered. Control animals received the same volume and schedule of bottled water. Animals received eight administrations over the four-week treatment period. The dosing schedule was selected based on previous experimental studies, and the administered volume was derived from the volume typically consumed in ritual settings (approximately 100 mL/70 kg) and from previous rodent studies [32,36,37].

### 4.3. Chemical Characterization of the B. caapi Extract

Tetrahydroharmine (THH), harmaline, and harmine concentrations were determined by high-performance liquid chromatography with diode-array detection (HPLC-DAD), as previously described [12]. Briefly, 200 µL of each sample was mixed with 800 µL methanol, vortexed, and centrifuged. A 200 µL aliquot of the supernatant was transferred to an autosampler vial containing 800 µL methanol. Chromatographic separation was performed using an Atlantis T3 column (150 × 3.0 mm, 3 µm) fitted with an Atlantis T3 guard column (30 × 10 mm, 5 µm; Waters Corporation, Milford, MA, USA). The mobile phases consisted of (A) 10 mmol/L phosphoric acid in ultrapure water (pH 3.0) and (B) acetonitrile, at a flow rate of 1 mL/min. The initial conditions (40% A/60% B) were maintained for 1 min, followed by a linear change to 5% A/95% B over 13 min. The measured concentrations were 380 µg/mL THH, 126 µg/mL harmaline, and 650 µg/mL harmine.

### 4.4. Experimental Design

**Figure 7:**
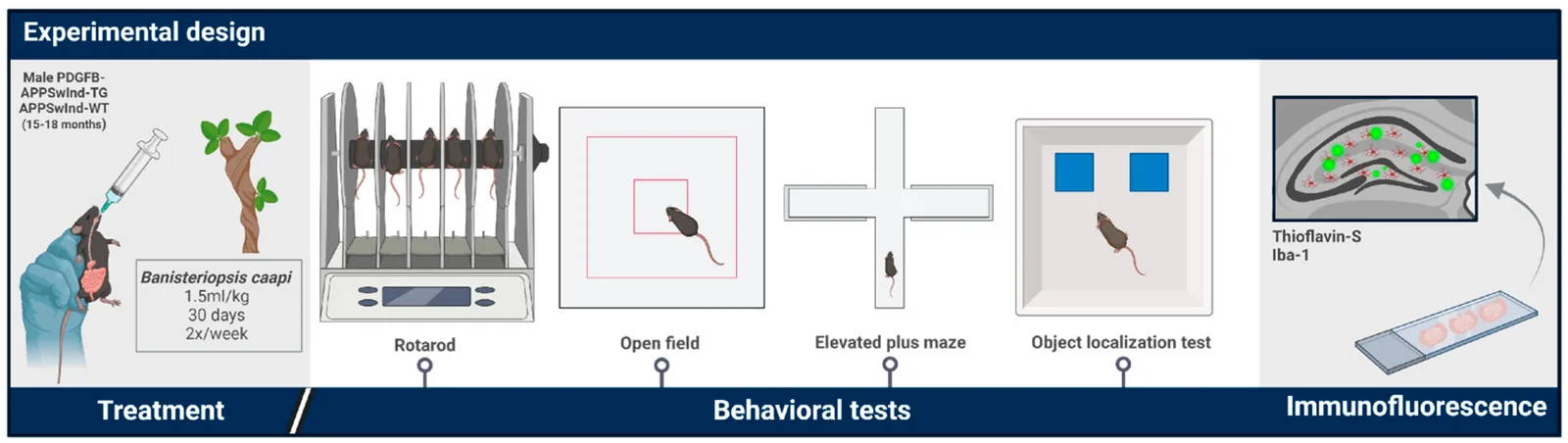
Experimental timeline. Animals received *B. caapi* extract (1.5 mL/kg) or vehicle by oral gavage twice weekly for four weeks, followed by behavioral testing and tissue collection. Created in BioRender. Calvelo graça, S. (2026) https://BioRender.com/d30b426 (accessed on 12 August 2026).

Following genotyping, aged male mice (15–18 months old) were separated according to genotype (WT or TG) and randomly assigned within each genotype to receive either vehicle or *B. caapi* treatment, resulting in four experimental groups: WT + vehicle, WT + *B. caapi*, TG + vehicle, and TG + *B. caapi*. Animals received *B. caapi* extract (1.5 mL/kg) or vehicle by oral gavage twice weekly for four consecutive weeks, totaling eight administrations. Behavioral assessments were conducted during the treatment period according to the experimental timeline shown in Figure 7. Behavioral assessments, histological image acquisition, and histological quantification were performed by investigators blinded to the experimental group assignments. At the end of the experimental period, animals were euthanized and brain tissue was collected for histological analyses. The number of animals used for each analysis is reported in the corresponding Results and figure legends. For histological analyses, the animal was considered the experimental unit, while multiple sections, images, plaques, or cells obtained from the same animal were treated as subsamples and were not considered independent biological replicates. No animals were excluded from the study after group allocation. For histological analyses, inclusion of tissue sections was based on tissue availability and technical suitability for the corresponding endpoint. The overall experimental design and sequence of procedures are summarized in Figure 7.

### 4.5. Behavioral Studies

Rotarod: Animals were acclimated to the Rotarod apparatus for mice (Insight EFF 412; Insight Equipamentos, Ribeirão Preto, SP, Brazil), which consists of a box measuring 43 cm in height, 40 cm in width and 34 cm in depth with a motorized cylinder where the animal is positioned. During the acclimation, the animal is placed in a bay of the apparatus where it remains for five minutes with no movement of the motorized cylinder. After 1 h, the animals are placed back in the apparatus where they remain for five minutes, at the lowest rotation speed of the apparatus (10 rpm), with unlimited chances of re-exposure if the animal falls from the bar during the five minutes. The test day (24h after the training session) also consists of five minutes of exposure, where the apparatus gradually reaches 37 rpm. The test was stopped when the animal fell from the rod. The latency (seconds) to fall from the rod was considered [38].

### 4.6. Object-Location (OL)

Animals were acclimated for three consecutive days for two minutes with the researcher who performed the experiment, this being an extremely important protocol for the behavior of the animals during the experiment. After this stage, the animals were handled by the researcher for three days for two minutes in the room where the experiment took place. In the three days following the acclimation, animals were habituated to the experimental box (40 × 40 cm) without the presence of any object for five minutes. On the following day, the animals were submitted to the training session (T0), which consisted of exploring the box for five minutes with the presence of two identical objects in the center line of the box. 90 min after the training exposure, the short-term memory test was assessed, where the animals were positioned in the box with one of the objects displaced, and allowed to explore for five minutes. Finally, 24 h after training, to assess long-term memory, the animals were positioned again in the box with the same conditions as the last exposure (one of the objects displaced from its original position at T0), and allowed to explore for five minutes (see scheme in Figure 4). Thus, the number of entries in each object (considered when the mouse points its snout directly at the object at a minimum distance of 2 cm) were considered [39].

The time difference index and entries difference index were calculated based on the time of exploration or entries in each object (see the calculation used for index data in the scheme of Figure 4). 

### 4.7. Elevated Plus Maze (EPM)

Anxiety-like behavior was assessed using the elevated plus-maze (EPM), which consisted of two open arms (30 × 5 cm) and two closed arms (30 × 5 × 10 cm), connected by a central platform (5 × 5 cm) and elevated 50 cm above the floor. Each mouse was placed on the central platform facing a closed arm and allowed to explore the apparatus for 5 min, while a camera positioned above the apparatus recorded the animal’s performance. The percentage of time spent in the open arms and the percentage of open-arm entries were quantified using Smart 3.0 software [40]. Head-dipping and risk-assessment behaviors were also recorded.

### 4.8. Open Field (OF)

Spontaneous locomotor activity was assessed in an open-field arena (40 × 40 cm). Each mouse was allowed to explore freely for 5 min. The arena was cleaned with 5% ethanol between animals. The experiment was recorded with a digital camera. Total distance traveled, mean speed, and time spent in the central zone were quantified and analyzed [11].

Behavioral sessions were video-recorded, and the corresponding behavioral parameters were analyzed using the Smart 3.0 video-tracking system (Panlab, Barcelona, Spain), as specified for each test above.

*Histological procedures and image analysis*: At the end of the experiments, the animals were deeply anesthetized with sodium pentobarbital (60 mg/kg) and perfused through the left cardiac ventricle with PBS (pH 7.4) followed by paraformaldehyde (4% in 0.1 M phosphate, pH 7.4). The brains were removed and stored in this fixative for 4 h at 4 °C. After this period, the material was transferred to a solution containing 30% sucrose in phosphate buffer (PB) for cryoprotection. After 24 h, the brains were cut in a microtome (SM2010 R, Leica Microsystems, Germany) at a thickness of 30 µm and stored in a cryoprotective solution (20% glycerol, 30% ethylene glycol in 50 mM phosphate, pH 7.4) until histological processing.

Free-floating sections were initially processed for Iba1 immunofluorescence. After incubation with 10% normal horse serum for 45 min, sections were incubated overnight with a primary antibody against Iba1 (1:500; Fujifilm Wako, Osaka, Japan), followed by an Alexa Fluor 594-conjugated anti-rabbit secondary antibody (1:500; Invitrogen, Carlsbad, CA, USA). To visualize fibrillar amyloid deposits, the same sections were subsequently incubated with 1% Thioflavin-S (T1892; Sigma-Aldrich, St. Louis, MO, USA) for 8 min and washed with 70% ethanol. Sections were then mounted in rostrocaudal sequence on gelatin-coated slides and coverslipped with DPX.

Histological analyses were performed in the dorsal hippocampus, approximately −1.34 to −2.06 mm from bregma, according to the neuroanatomical nomenclature of Paxinos and Franklin’s mouse brain atlas [41]. One of every six 30 μm coronal sections was analyzed, providing a 180 μm interval between sampled sections. Images were acquired using a Leica DMi8 microscope (Leica Microsystems, Wetzlar, Germany) under identical acquisition settings across experimental groups. Magnifications of 5× and 10× were used for anatomical documentation, whereas 20× and 40× images were used for quantitative analyses.

Hippocampal amyloid pathology was evaluated in four animals per experimental group. For plaque quantification, ThS-positive plaques were automatically counted in four hippocampal sections, with both the left and right hemispheres analyzed (eight fields per animal) using ImageJ (version 1.41; National Institutes of Health, Bethesda, MD, USA). The number of ThS-positive plaques was determined for each field, and the mean plaque number across the eight analyzed fields was calculated for each animal. The ThS-positive area was quantified using a threshold-based analysis in ImageJ, with identical threshold settings applied to all images. For each animal, the ThS-positive area was measured in four hippocampal sections, with both the left and right hemispheres analyzed (eight fields per animal). ThS-positive area (µm^2^) was determined for each field, and the values from the eight fields were averaged to obtain a single mean ThS-positive area value for each animal. To characterize plaque size distribution, the area of each individual ThS-positive plaque was measured in µm^2^. For each animal, the individual plaque areas obtained from all analyzed fields were pooled within that animal, and the median plaque area was calculated. Thus, a single median plaque-area value was obtained for each animal and used as the experimental unit for statistical analysis.

Plaque-associated microglia were evaluated in three 40× images of the dorsal hippocampus acquired from each animal using the same anatomical sampling criteria across groups. Twelve Thioflavin-S-positive plaques were evaluated per animal, and the number of Iba1-labeled cells immediately adjacent to each plaque was manually quantified. The values obtained from the 12 plaques were averaged to generate the animal-level value used for statistical analysis.

Iba1-labeled cells were analysed using threshold-based image analysis in ImageJ. The threshold was applied to the Iba1-labeled images to identify the cellular profiles within the predefined regions of interest, and the number of Iba1-labeled cells was quantified for each analyzed field, allowing the number of labeled Iba1 cells to be quantified using an automated procedure rather than by manual counting. For morphological analysis, the thresholded Iba1-labeled microglial profiles were converted into binary images and subjected to skeletonization using the Analyze Skeleton plugin to determine endpoints and total process length. 

Iba1-labeled cell quantification and skeletonization in the dorsal hippocampus were assessed in four animals per experimental group. Two 20× images were acquired from each hemisphere, resulting in four images per animal. Process length and endpoints values were normalized to the number of Iba1-labeled cells. Values obtained from the four images were averaged to generate a single representative value for each animal. CA1-specific microglial analyses were performed in three animals per experimental group, as anatomically suitable sections for quantitative evaluation of this subfield were available for three animals in each group. Three 40× images were acquired from each hemisphere, resulting in six images per animal. Iba1-labeled cell quantification, endpoints, and process length were analysed using the same procedures described above, and measurements obtained from the six images were averaged to generate a single value for each animal. No animals or histological samples were excluded from the dorsal hippocampal analyses; the smaller sample size used for the CA1-specific analysis resulted solely from the availability of anatomically suitable sections.

Throughout the histological analyses, images, sections, individual cells, and individual plaques were considered subsamples rather than independent experimental units. Measurements obtained from these subsamples were therefore aggregated at the animal level, and the individual animal was considered the experimental unit for statistical analysis.

### 4.9. Statistical Analysis

No single primary outcome was prespecified; behavioral, fibrillar amyloid, and microglial measures were evaluated as complementary experimental outcomes. All data are presented as mean ± standard error of the mean (SEM). Behavioral parameters from the object location task (time discrimination index, entry discrimination index, total distance traveled, and total exploration time) were analyzed using a three-way mixed-effects model (REML), with genotype (WT or TG) and treatment (vehicle or *B. caapi*) as between-subject factors and time (T0, 90 min, and 24 h) as the within-subject repeated-measures factor. For the object-location discrimination indices, statistical analyses were performed to assess differences between experimental groups and/or time points. Although zero represents the theoretical chance level for this measure, the observed values were not statistically compared with zero. Accordingly, the analysis was restricted to between-group comparisons and was not used to infer the presence or absence of impairment in global cognitive function. When the assumption of sphericity was violated, the Geisser–Greenhouse correction was applied. Significant main effects or interactions were followed by Sidak’s multiple-comparisons test.

Body weight was expressed as the percentage of baseline body weight (Week 1 = 100%). Because baseline values were normalized to 100% for all animals, statistical analyses were performed from Week 2 onward using a linear mixed-effects model, with genotype, treatment, and time as fixed effects and animal as a random effect. Pairwise comparisons were performed using Sidak’s multiple-comparisons test when appropriate.

Histological measurements were analyzed using two-way analysis of variance (ANOVA), with genotype (WT or TG) and treatment (vehicle or *B. caapi*) as fixed factors. The individual animal was considered the experimental unit for all histological analyses. Measurements obtained from multiple sections, images, plaques, or cells from the same animal were treated as subsamples and were averaged to generate a single value per animal for each outcome before statistical analysis. Total Thioflavin-S-positive area and animal-level median plaque area were compared between TG + Vehicle and TG + *B. caapi* mice using two-tailed unpaired *t*-tests. 

Statistical significance was set at *p* ≤ 0.05. Analyses performed using two-way ANOVA were conducted in GraphPad Prism version 8.0.1 (GraphPad Software, San Diego, CA, USA), whereas linear mixed-effects models were performed in R (R Foundation for Statistical Computing, Vienna, Austria) using the lme4, lmerTest, and emmeans packages.

## 5. Conclusions

In conclusion, chronic intermittent administration of *Banisteriopsis caapi* in aged PDGFB-APPSwInd mice did not consistently modify the behavioral or microglial parameters evaluated under the experimental conditions employed. In contrast, treatment was associated with an increased number of hippocampal Thioflavin-S-positive fibrillar amyloid plaques in TG mice, without corresponding changes in total ThS-positive area or median plaque size. Microglial structural alterations were predominantly associated with the transgenic phenotype, particularly in the CA1 region, and were not significantly modified by *B. caapi* treatment. Together, these findings indicate that repeated *B. caapi* exposure may differentially influence components of amyloid pathology without producing parallel changes in the behavioral and microglial outcomes assessed. The present study therefore expands the experimental evidence regarding the biological effects of chronic intermittent *B. caapi* exposure in the context of established amyloid pathology and provides a basis for further investigation across different stages of disease progression.

## Data Availability

The data presented in this study are available on request from the corresponding author due to study’s dataset is being maintained within the institutional research repository and contains raw image-analysis files requiring contextual.

## Abbreviations

The following abbreviations are used in this manuscript:

Aβ	Amyloid-beta
AD	Alzheimer’s Disease
ANOVA	Analysis of Variance
APP	Amyloid Precursor Protein
*B. caapi*	*Banisteriopsis caapi*
CA1	Cornu Ammonis 1
CA2	Cornu Ammonis 2
CA3	Cornu Ammonis 3
CNS	Central Nervous System
COX-2	Cyclooxygenase-2
DMT	N,N-Dimethyltryptamine
EPM	Elevated Plus Maze
GABA	Gamma-Aminobutyric Acid
GABAA	Gamma-Aminobutyric Acid Type A Receptor
HPLC-DAD	High-Performance Liquid Chromatography with Diode Array Detection
Iba1	Ionized Calcium-Binding Adapter Molecule 1
iNOS	Inducible Nitric Oxide Synthase
LPS	Lipopolysaccharide
MAO-A	Monoamine Oxidase A
MAO-B	Monoamine Oxidase B
NF-κB	Nuclear Factor Kappa B
NFT	Neurofibrillary Tangles
OF	Open Field
OL	Object Location
PBS	Phosphate-Buffered Saline
PB	Phosphate Buffer
PRRs	Pattern Recognition Receptors
RAGE	Receptor for Advanced Glycation End Products
REML	Restricted Maximum Likelihood
SEM	Standard Error of the Mean
TG	Transgenic
THH	Tetrahydroharmine
ThS	Thioflavin-S
TLR	Toll-Like Receptor
TNF-α	Tumor Necrosis Factor Alpha
TREM-2	Triggering Receptor Expressed on Myeloid Cells 2
WT	Wild Type
